# Draft genome sequences of four Rhizobium spp. isolates, including one potential new species, from tropical legume plants in Costa Rica

**DOI:** 10.1099/acmi.0.001012.v3

**Published:** 2026-04-02

**Authors:** Donifer Campos-Parra, Andres Blanco-Picado, Jimena Herrera-Quesada, Bradd Mendoza-Guido, Keilor Rojas-Jimenez

**Affiliations:** 1 Escuela de Biología, Universidad de Costa Rica, Sede Rodrigo Facio Brenes, 11501-2060 Montes de Oca, San José, Costa Rica; 2 Instituto de Investigaciones en Salud (INISA), Universidad de Costa Rica, Sede Rodrigo Facio Brenes,, 11501-2060 Montes de Oca, San José, Costa Rica

**Keywords:** *Fabaceae*, nitrogen fixation, nodulation, *Rhizobiaceae*, symbiosis

## Abstract

We present the draft genomes of four strains of *Rhizobium* isolated from root nodules of *Cojoba arborea*, *Lonchocarpus felipei*, *Mimosa pigra* and *Calliandra haematocephala* in Costa Rica. Through comparative genomics, including the estimation of the average nucleotide identity (ANI), digital DNA–DNA hybridization (dDDH) and phylogenomic analysis, we determined that *Rhizobium* sp*.* LEGMi-135b represents a potentially novel species near *Rhizobium hainanense* (ANI: 94%; dDDH: 53.2%). The strain LEGMi-12c was associated with *R. hainanense* (ANI: 98%; dDDH: 87%), strain LEGMi-166a to *Rhizobium altiplani* (ANI: 100%; dDDH: 99.3%) and strain LEGMi-198b was related to *Rhizobium cerradonense* (ANI: 98%; dDDH: 81.4%). Using functional annotation tools, we also determined *in silico* the presence of genes related to nodulation and nitrogen fixation, such as *fix*, *nif*, *nod*, *nol* and *ntr*, in each strain. With this work, we provide valuable genomic resources of the genus *Rhizobium* that will be useful for future studies on rhizobial taxonomy, symbiosis and ecology in tropical ecosystems.

## Data Summary

The raw read sequences of the four *Rhizobium* spp. isolates have been deposited in GenBank under the BioProject accession no. PRJNA1084876 and the Sequence Read Archive (SRA) accession no. SRP493729. The sample accession details are as follows: *Rhizobium* sp. LEGMi-12c (BioSample: SAMN44800730; Genome accession: GCF_054114145.1), *Rhizobium* sp. LEGMi-135b (BioSample: SAMN44800728; Genome accession: GCF_054114165.1), *Rhizobium* sp. LEGMi-166a (BioSample: SAMN46966679; Genome accession: GCF_054114185.1) and *Rhizobium* sp. LEGMi-198b (BioSample: SAMN44800729; Genome accession: GCF_054114205.1). The 16S rRNA gene sequences of the isolates were submitted to the National Center for Biotechnology Information (NCBI) GenBank database and assigned the following accession numbers: PP439182 (LEGMi-12c), PP439210 (LEGMi-135b), PP439229 (LEGMi-166a) and PP439245 (LEGMi-198b).

## Announcement

Bacteria of the genus *Rhizobium* are key nitrogen-fixing symbionts of leguminous plants; yet, the diversity of *Rhizobium* and other rhizobia associated with many tropical legumes remains underexplored, suggesting that numerous taxa and symbiotic associations remain undescribed [1,3]. Symbiotic *Rhizobium* species often display high genetic variation in nodulation and nitrogen fixation genes (e.g. *fix*, *nif*, *nod*, *nol* and *ntr*), even within the same species. Therefore, resolving both species identity and symbiotic genes is crucial for understanding host specificity and inter-strain variation [1]. Notably, other studies have reported that *nod* gene sequences clustered by bacterial core-genome relatedness or by geographic origin rather than host taxonomy, indicating horizontal transfer of symbiotic genes and a mosaic distribution of host-range determinants [4].

Here, we isolated and characterized the genomes of *Rhizobium* strains from tropical legume hosts cultivated in Costa Rica and placed them within a species framework. We clarify their relationships to *Rhizobium hainanense* and *Rhizobium altiplani*, frequent closest matches for Latin American isolates, testing whether these tree-associated strains represent adjacent but potentially under-sampled lineages [5]. This work extends the ecological and biogeographic scope of *Rhizobium* beyond crop-dominated datasets [5].

We sampled root nodules from four tropical tree species cultivated in Costa Rican nurseries: *Cojoba arborea*, *Lonchocarpus felipei*, *Mimosa pigra* and *Calliandra haematocephala*. Small plants (30–100 cm tall, 1–3 years old) were selected. Roots were manually examined for nodules, and nodule subsamples (1–4, depending on size) were collected for bacterial isolation. Sampling was conducted under the corresponding local and institutional permits from the University of Costa Rica.

Upon arrival at the laboratory, we transferred the nodules to 2 ml tubes and rinsed them twice with sterile distilled water. We then surface-disinfected the nodules with 70% ethanol for 1 min, followed by two sterile distilled water rinses. To ensure sterility, we treated the nodules with 2% sodium hypochlorite for 5 min and rinsed them seven times with sterile distilled water. We aseptically transferred the disinfected nodules to new tubes containing 250 µl of sterile PBS (pH 7.4). We macerated the root nodules using autoclaved plastic pistils to release the bacterial contents. We serially diluted the resulting suspension ten-fold (10⁻¹ and 10⁻²) in PY broth (peptone-based medium) containing 5 g l^−1^ peptone, 1 g l^−1^ anhydrous dextrose, 0.5 g l^−1^ dipotassium phosphate, 0.2 g l^−1^ magnesium sulphate, 0.1 g l^−1^ sodium chloride.

We spread 50 µl aliquots from each dilution onto PY-Agar plates supplemented with cycloheximide (40 mg l^−1^) to inhibit fungal growth. We incubated the plates at 28 °C for up to 3 weeks. We picked individual colonies and streaked them onto fresh PY-Agar plates with cycloheximide. Finally, we cryopreserved the purified strains (LEGMi-12c, LEGMi-135b, LEGMi-166a and LEGMi-198b) in PY broth with 20% glycerol and stored them at −80 °C in the collection of the Laboratory of Genetics and Ecology of Micro-organisms (LEGMi) at the School of Biology, University of Costa Rica. All laboratory work with environmental *Rhizobium* isolates followed the institutional biosafety guidelines for organisms in Risk Group 1.

To identify potential novel rhizobial species, we cultured the isolates and extracted DNA using the DNeasy PowerSoil Kit (Qiagen), following the manufacturer’s instructions. Whole-genome sequencing libraries were constructed with the Illumina DNA Prep kit. Briefly, genomic DNA underwent random fragmentation, followed by end repair, A-tailing and ligation to Illumina-compatible adapters. Libraries were subsequently PCR-enriched, size-selected, purified and evaluated for quality and quantity using Qubit fluorometry, real-time PCR and an Agilent Bioanalyzer. Quantified libraries were pooled, and paired-end whole-genome sequencing (2×150 bp) was conducted by Novogene (CA, USA) on an Illumina NovaSeq PE150 platform, except for isolate LEGMi-135b, which was sequenced using both Illumina and PacBio technologies.

Illumina raw reads were processed with fastp v0.20.1 (default parameters, Q30 threshold), and high-quality reads were assembled in SPAdes (v.3.15.4) using k-mers of 21, 33, 55 and 77 [6,7]. Strain LEGMi-135b was assembled using a hybrid Illumina–PacBio approach implemented in SPAdes. Short Illumina reads and long PacBio reads were jointly assembled using the hybridSPAdes pipeline, which integrates long-read information to resolve repeats and close gaps in short-read assemblies via the exSPAnder module [8]. The assembly was performed using k-mer sizes of 33, 55, 77, 99 and 127. Contigs <1,000 bp were removed using seqtk, and assembly statistics were generated with Quast [9,10]. Genome completeness and contamination were assessed using CheckM2 v1.0.1 [11]. For genome annotation, we employed eggNOG-mapper v2.1.6 and RAST tools [12,13]. All *Rhizobium* isolates contained *fix*, *nif*, *nod*, *nol* and *ntr* genes, suggesting *in silico* nodulation and nitrogen fixation potential (Table 1).

**Table 1.** Genome features of the four *Rhizobium* spp. isolates

Strain characteristic	Data for:			
	*Rhizobium* sp. -LEGMi-12c	*Rhizobium* sp. -LEGMi-135b	*Rhizobium* sp. -LEGMi-166a	*Rhizobium* sp. -LEGMi-198b
Legume host species	*C. arborea*	*L. felipei*	*M. pigra*	*C. haematocephala*
No. of reads (post-quality control)	8,188,458	10,503,224	9,500,990	8,669,270
No. of contigs	119	33	107	22
No. of genes	6,454	6,192	7,464	6,357
Genome length (bp)	6,846,952	6,620,416	7,565,831	6,828,554
Coverage (×)	179.39	237.97	210.30	190.43
N50 (bp)	109,404	571,440	185,397	817,417
G+C content (mol%)	59.82	59.77	59.48	59.53
Completeness	99.94	99.98	100	100
Contamination (%)	0.47	0.15	2	0.18
Closest 16S rRNA match – accession	*Rhizobium* sp. strain IITA-TZ190 -OM909439	*Rhizobium* sp. strain IITA-TZ190 -OM909439	*R. altiplani* strain BR10893 -OM144531	*R. rhizogenes* strain SQL8 -MN480505
Nodulation genes (eggNOG-mapper per annotations)	*nodL*, *nodN*,* nodQ*, *nodT* and *nodW*	*nodL*, *nodN*, *nodQ*, *nodT* and *nodW*	*nodA*, *nodB*, *nodC*, *nodC*, *nodD*, *nodH*, *nodI*, *nodJ*, *nodN*, *nodQ*, *nodS*, *nodU* and *nodZ*	*nodA*, *nodB*, *nodC*, *nodD*, *nodD1*, *nodI*, *nodJ*, *nodL*, *nodN*, *nodQ*, *nodS*, *nodT*, *nodU* and *nolO*
Nitrogen fixation genes (eggNOG-mapper annotations)	*fixK and nifU*	*fixK and nifU*	*fixA*, *fixB*, *fixC*, *fixG*, *fixH*, *fixI*, *fixJ*, *fixK*, *fixQ*, *fixR*, *fixS*, *fixU*, *fixX*, *nifA*, *nifB*, *nifD*, *nifE*, *nifH*, *nifK*, *nifN*, *nifQ*, *nifS*, *nifU*, *nifW*, *nifX* and *nifZ*	*fixA*, *fixB*, *fixC*, *fixG*, *fixH*, *fixI*, *fixJ*, *fixK*, *fixL*, *fixQ*, *fixS*, *fixU*, *fixX*, *nifA*, *nifB*, *nifD*, *nifE*, *nifH*, *nifK*, *nifN*, *nifQ*, *nifS*, *nifT*, *nifU*, *nifX* and *nifZ*
Regulatory genes (eggNOG-mapper annotations)	*nolR*, *ntrB*, *ntrC*, *ntrX* and *ntrY*	*nolR*, *ntrB*, *ntrC*, *ntrX* and *ntrY*	*nolR*, *ntrB*, *ntrC*, *ntrX* and *ntrY*	*nolR*, *nolR*, *ntrB*, *ntrC*, *ntrX* and *ntrY*

To identify related species, we initially used the Type Strain Genome Server (TYGS) to perform whole-genome comparisons based on digital DNA–DNA hybridization (dDDH) [14]. In parallel, the Genome Taxonomy Database Toolkit (GTDB-Tk) was used to assign standardized taxonomic classifications according to the Genome Taxonomy Database (GTDB) [15]. For each genome, GTDB-Tk assigned a standardized taxonomic classification based on phylogenomic placement within the GTDB bac120 reference tree and on average nucleotide identity (ANI) comparisons to GTDB reference genomes using FastANI software [15]. The FastANI reference genome and its associated species radius were used to support species delimitation (Table 2).

**Table 2.** GTDB-Tk genome-based taxonomic classification of the analysed strains

Strain	Classification	FastANI reference	FastANI reference radius
LEGMi-166a	*R. altiplani*	GCF_001542405.1	95
LEGMi-198b	*Rhizobium* sp002944315	GCF_002944315.1	95
LEGMi-12c	*R. hainanense*	GCF_900094555.1	95
LEGMi-135b	*Rhizobium* sp.	–*	–

* –: No FastANI reference genome was assigned because no ANI value met the species-level threshold.

Phylogenomic reconstruction was performed using the bac120 marker gene set, which comprises 120 conserved single-copy bacterial genes. The resulting reference phylogeny was subsequently pruned to obtain a sub-tree containing the genomes analysed in this study together with their closest GTDB reference genomes, and this sub-tree was used for further phylogenetic interpretation (Fig. S1, available in the online Supplementary Material).

Based on the closest related species identified through these analyses, a list of GenBank accession numbers corresponding to *Rhizobium* genomes was manually compiled. This process included a comprehensive literature search to review the taxonomy and reported closest relatives of these species, ensuring the inclusion of all available type strains and of the closest related species with sequenced genomes. Then, we performed further analysis using GenFlow, an in-house pipeline that automates genome processing and phylogenomic analysis [16]. GenFlow retrieves user-specified reference genomes from GenBank, renames them and processes them in Anvi'o (v.7.1) [17]. CDSs were annotated with Prodigal, and shared core single-copy genes were extracted [18].

**Fig. 1.**
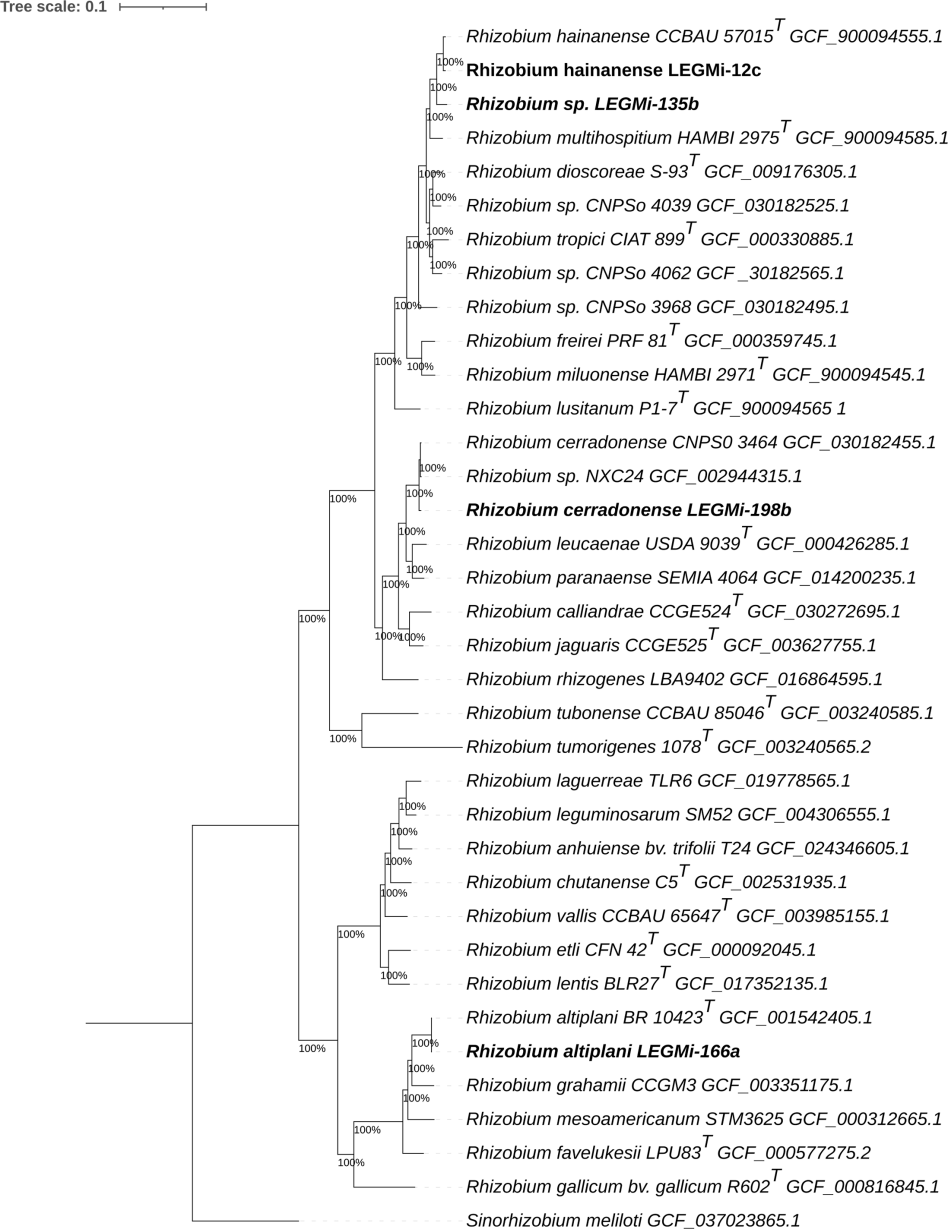
A phylogenomic tree of the core genomes from rhizobial isolates generated using FastTree with the JTT+CAT model and 1,000 bootstrap replicates. The analysis included 37 genomes, and a total of 1,294 single-copy orthologous gene sequences were used to build the concatenated alignment for tree inference. Reference genomes included in the phylogenetic analyses were manually selected from the GenBank database, prioritizing type strains and ensuring the incorporation of the closest available *Rhizobium* genomes identified through TYGS results, published literature and, when possible, designated reference genomes from type material. The genome from *Sinorhizobium meliloti* was used as the outgroup. The phylogenetic tree was visualized using iTOL v6. Genome accession numbers are displayed next to each strain. Type strains are indicated by the superscript 'T'. Bootstrap values are displayed at the nodes, and the four putative *Rhizobium* species are highlighted in bold.

We aligned amino acid sequences of these genes with MAFFT v7.397 and obtained the resulting concatenated protein alignment from the 1,294 single-copy core gene clusters comprising 16,520,759 amino acid positions [19]. We constructed a phylogenetic tree using FastTree with the JTT+CAT model; the resulting bootstrap support percentages for key nodes were all 100% (Fig. 1) [20].

**Fig. 2.**
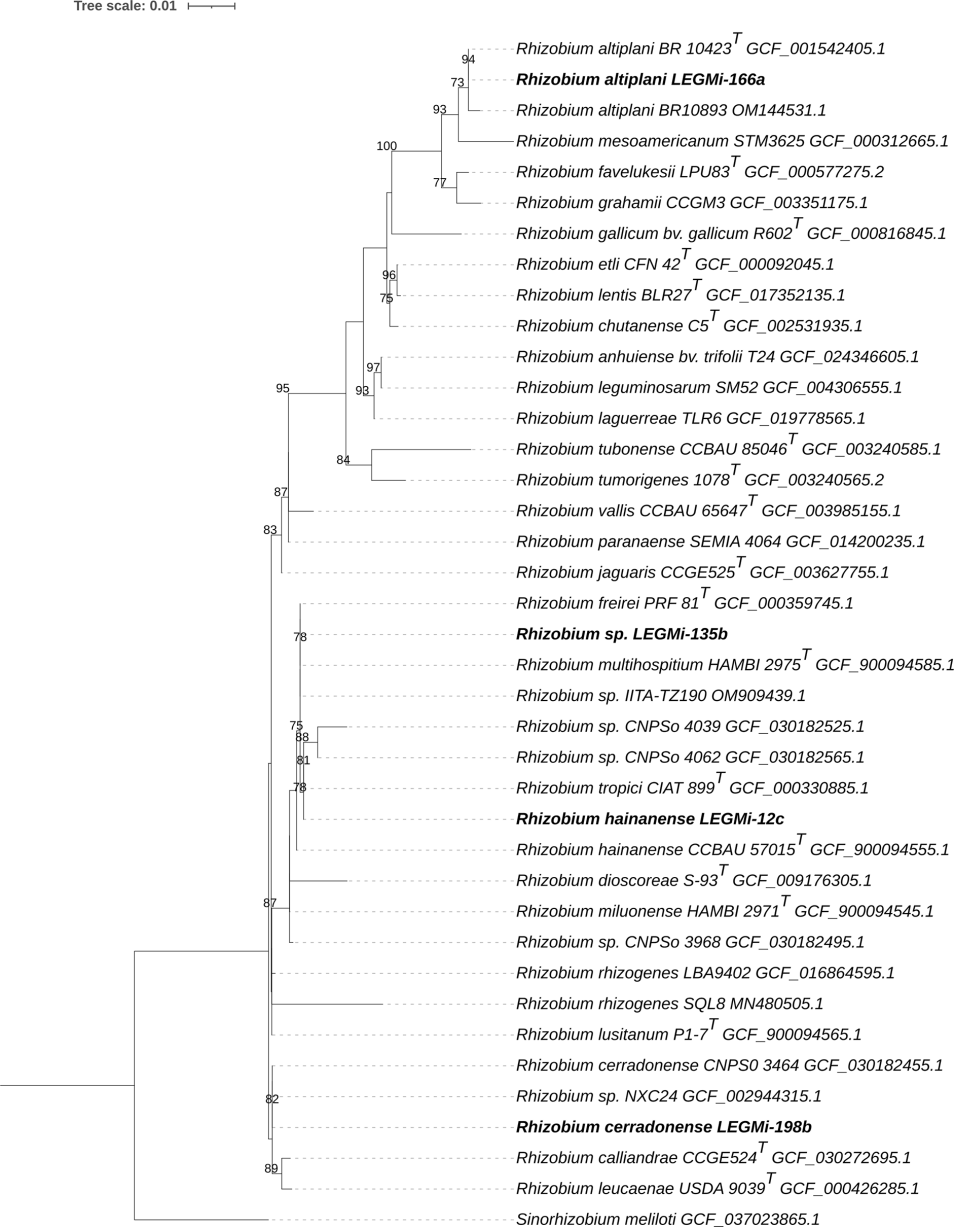
A maximum-likelihood 16S rRNA gene phylogenetic tree from rhizobia isolates was constructed using IQ-TREE v2.2.0 with the TN+F+I+G4 model and 1,000 bootstrap resamples. This tree represents the phylogeny of 37 genes from species closely related to our isolates, as determined by TYGS, blastn and a literature review. *Sinorhizobium meliloti* was used as an outgroup. The phylogenetic tree was visualized using iTOL v6. Reference genomes incorporated into the tree were manually chosen from the GenBank database. Genome accession numbers are displayed next to each strain. Type strains are indicated by the superscript 'T'. Bootstrap values ≥70% are displayed at the nodes, and the four putative *Rhizobium* species are highlighted in bold.

For the construction of the 16S rRNA gene phylogeny, 16S rRNA sequences were retrieved from the genomes using the *anvi-get-sequences-for-hmm-hits* tool in Anvi’o v7.1, which utilizes HMMER v3.3.2 for gene detection (Fig. 2) [21]. For the following strain, *Rhizobium* sp. IITA-TZ190 (OM909439), *Rhizobium* sp. IITA-TZ190 (OM909439), *R. altiplani* BR10893 (OM144531) and *Rhizobium rhizogenes* SQL8 (MN480505), genome accession numbers were not available in the National Center for Biotechnology Information (NCBI) database. Therefore, we retrieved their 16S rRNA gene sequences from the NCBI database and aligned them with the 16S sequences extracted from the available *Rhizobium* genomes. Sequence alignment was performed using MAFFT v7.397, and a maximum-likelihood phylogeny was inferred with IQ-TREE v2.2.0 [22] under the TIM3 +F+I+G4 substitution model with 1,000 bootstrap replicates. The phylogenomic trees were visualized using iTOL (v.6.9.1) [23].

To assess the genomic relatedness between the studied isolates and their closest relatives, dDDH was estimated using the Genome-to-Genome Distance Calculator 3.0 (formula 2, d4) (Table 3) [24]. Additionally, average nucleotide identity (ANI) values were calculated using pyANI software (Table 4) [25].

**Table 3.** dDDH (d4) values (in %) between *Rhizobium* strains under study and their closest published species

	*R. hainanense* CCBAU- 57015^T^- GCF_900094555.1	*R. altiplani* BR 10423^T^- GCF_001542405.1	*R. cerradonense* CNPSo 3464- GCF_030182455.1
*Rhizobium* sp.-LEGMi-12c	87.00	21.20	25.60
*Rhizobium* sp.*-*LEGMi-135b	53.20	21.50	25.50
*Rhizobium* sp*.-*LEGMi-166a	21.10	99.30	21.60
*Rhizobium* sp.*-*LEGMi-198b	25.60	21.70	81.40

**Table 4.** ANI values between *Rhizobium* strains under study and their closest published species

	*R. hainanense* CCBAU- 57015^T^- GCF_900094555.1	*R. altiplani* BR 10423^T^- GCF_001542405.1	*R. cerradonense* CNPSo 3464- GCF_030182455.1
*Rhizobium* sp.-LEGMi-12c	98	84	86
*Rhizobium* sp.*-*LEGMi-135b	94	84	86
*Rhizobium* sp*.-*LEGMi-166a	84	100	85
*Rhizobium* sp.*-*LEGMi-198b	86	84	98

Based on genomic analyses, we conclude that strain LEGMi-135b represents a potentially new species, as it shows ANI and dDDH values below the commonly accepted species thresholds (95–96% for ANI and 70% for dDDH) when compared with the closest described *Rhizobium* type strains. This conclusion is further supported by the phylogenomic analysis, in which strain LEGMi-135b clusters near *R. hainanense* but on a distinct branch. In contrast, strains LEGMi-12c, LEGMi-198b and LEGMi-166a were closely related to *R. hainanense*, *Rhizobium cerradonense* and *R. altiplani*, respectively.

The identification of *fix*, *nif*, *nod*, *nol* and *ntr* genes through genomic *in silico* analyses suggests that these isolates possess the genetic potential for nodulation and nitrogen fixation in legumes. Previous studies have shown that phylogenomic analyses of *nod* genes can help predict potential nodulation capabilities and group strains into symbiovars with similar host ranges, although these inferences should be confirmed through *in vivo* nodulation assays [26,30]. Therefore, although the genomic content is consistent with nodule-associated lifestyles, functional validation through plant inoculation assays, gene expression analyses and biochemical tests remains necessary. Further studies should focus on the phenotypic and metabolic characterization of isolates, as well as determining their potential classification as symbiovars.

## Supplementary material

10.1099/acmi.0.001012.v3 Uncited Fig. S1.

## Abbreviations

ANI: average nucleotide identity
dDDH: digital DNA–DNA hybridization
GTDB: Genome Taxonomy Database
LEGMi: Laboratory of Genetics and Ecology of Micro-organisms
NCBI: National Center for Biotechnology Information
SCCGs: shared core single-copy genes
SRA: Sequence Read Archive
TYGS: Type Strain Genome Server
