# Supplementary material for: Draft genome sequences of four Rhizobium spp. isolates, including one potential new species, from tropical legume plants in Costa Rica
Source: Access Microbiol. 2026 Apr 2;8(4):001012.v3. doi: 10.1099/acmi.0.001012.v3 (PMC13046423; doi:10.1099/acmi.0.001012.v3)

Fig. S1. Subtree extracted from the GTDB-Tk bac120 phylogenomic tree, based on 120 conserved single-copy bacterial marker genes. Node support values correspond to those inferred by GTDB-Tk during phylogenomic reconstruction. Genome accession numbers are displayed next to each strain. The four putative Rhizobium species are highlighted in bold.

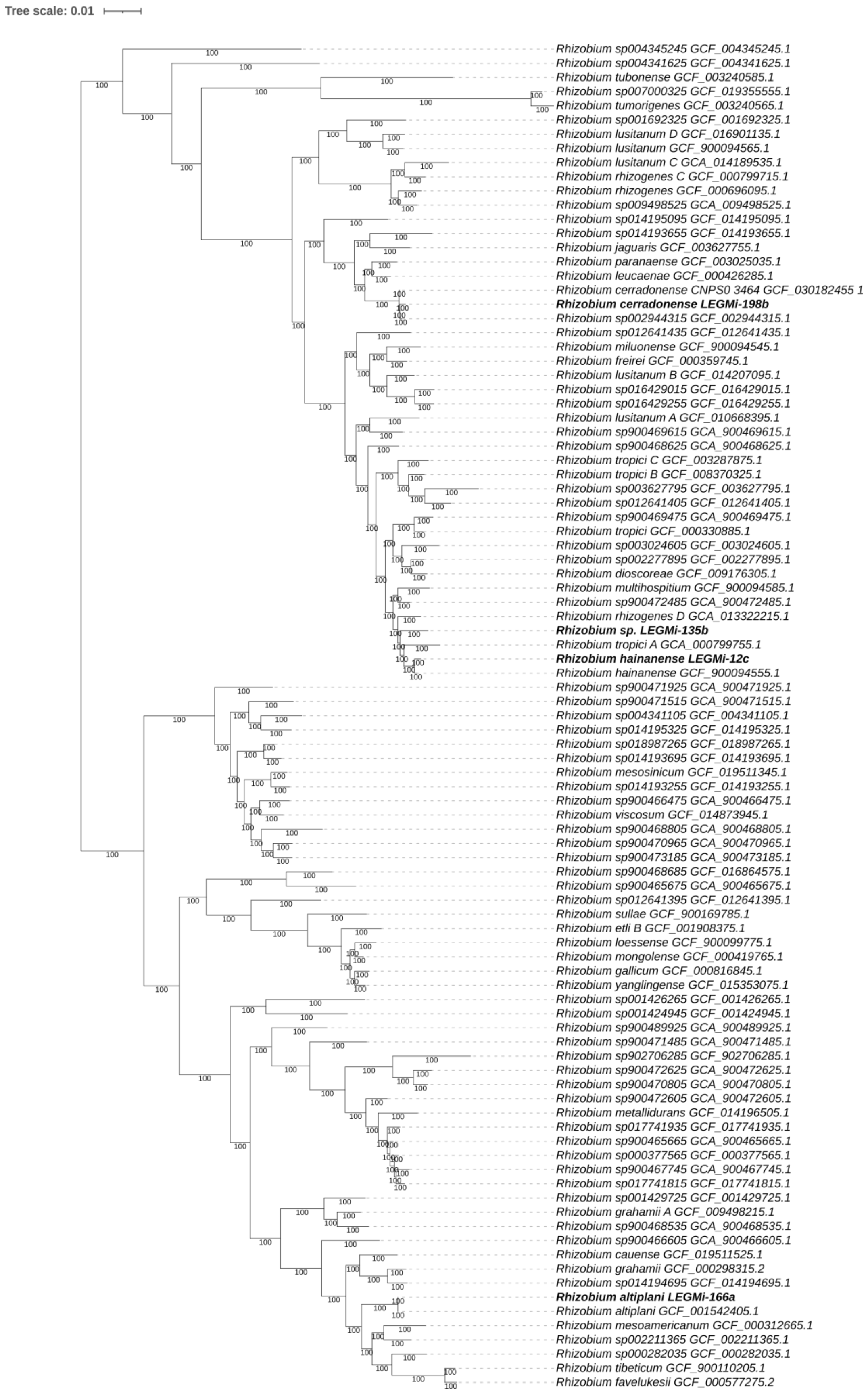

Supplement: Uncited Fig. S1. [file acmi-8-01012-s001.pdf]
